# Correction to: Microbiomes attached to fresh perennial ryegrass are temporally resilient and adapt to changing ecological niches

**DOI:** 10.1186/s40168-021-01122-w

**Published:** 2021-08-10

**Authors:** Sharon A. Huws, Joan E. Edwards, Wanchang Lin, Francesco Rubino, Mark Alston, David Swarbreck, Shabhonam Caim, Pauline Rees Stevens, Justin Pachebat, Mi-Young Won, Linda B. Oyama, Christopher J. Creevey, Alison H. Kingston-Smith

**Affiliations:** 1 Institute of Global Food Security, School of Biological Sciences, Queen’s University Belfast, 19 Chlorine Gardens, Belfast, Northern Ireland BT9 5DL UK; 2 Institute of Biological, Environmental and Rural Sciences, Aberystwyth University, Aberystwyth, SY23 3FG UK; 3 Laboratory of Microbiology, Wageningen University & Research, 6708 Wageningen, WE Netherlands; 4 Current work address: Palital Feed Additives, Velddriel, Netherlands; 5 Earlham Institute, Norwich, NR4 7UH UK; 6 Quadram Institute, Norwich, NR4 7UA UK

**Correction to: Microbiome 9, 143 (2021)**

**https://doi.org/10.1186/s40168-021-01087-w**

Following the publication of the original article [1], it was noticed that the figure image of Fig. 6 should be for Fig. 3. The image for Fig. 3 should be for Fig. 5 and Fig. 6 was missing. The correct Fig. 6 have been provided below and the original article has been updated to correct Figs. 3, 5 and 6.

**Fig. 6:**
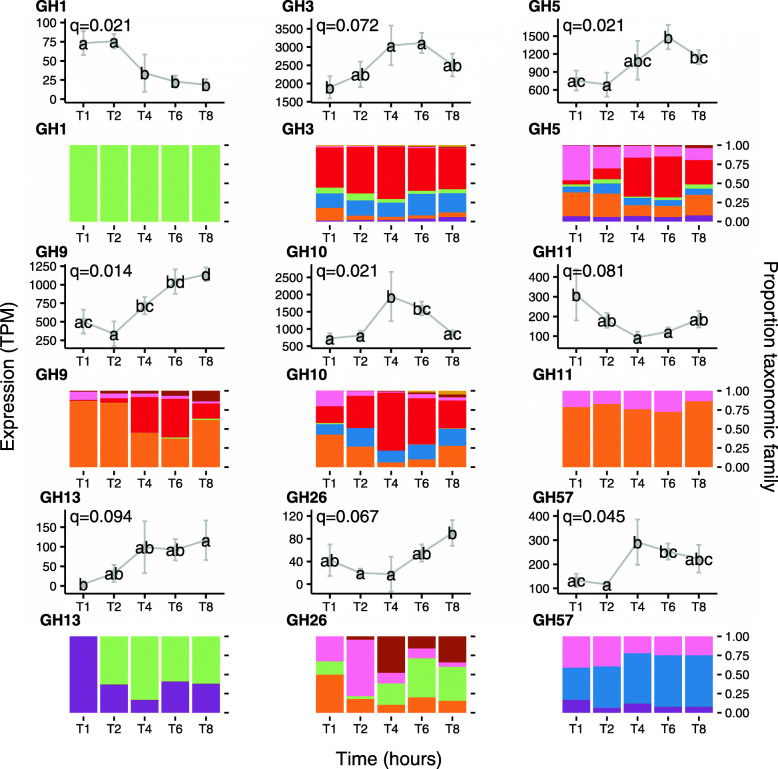
In-depth analysis of the temporal expression of differentially expressed carbohydrate-active enzyme (CAZymes, also known as glycosyl hydrolases (GH)) expressed genes by prokaryotes attached to fresh perennial ryegrass incubated within the rumen that differed significantly in their expression profile over rumen incubation time (line plots) and their respective taxonomic origins (bar chart below the corresponding line plot). Incubation time is indicated on the axis of the plots, i.e. T1 indicates an incubation time of 1 h. Brown bars: family Eubacteriaceae (genus *Eubacterium*); Pink bars: family Fibrobacteriaceae (genus *Fibrobacter*); Red bars: family Lachnospiraceae (genera *Butyrivibrio* and *Pseudobutyrivibro*); Blue bars: family Prevotellaceae (genus *Prevotella*); orange bars: Ruminococcaceae (genus *Ruminococcus*); Purple bars: Spirochaetaceae (genus *Treponema*). The significance of rumen incubation time on gene expression is indicated on each plot, with timepoint that significantly differ denoted by a different letter in the line plot
